# The analytic content is not semiadditive

**DOI:** 10.1007/s13324-024-00994-z

**Published:** 2024-11-28

**Authors:** Eduardo S. Zeron, Paul M. Gauthier

**Affiliations:** 1 Departamento de Matemáticas, Cinvestav del IPN, Apartado Postal 14-740, 07000 Ciudad de México, CDMX México; 2 Département de Mathématiques et de Statistique, Université de Montréal, Case postale 6128, Montréal, QC H3C 3J7 Canada

**Keywords:** Analytic content, Uniform approximation, Rational functions, 30E10, 30C10, 30H50

## Abstract

We show that the analytic content $\lambda (\cdot )$ is neither subadditive nor semiadditive. To be precise, for compact sets *K* in the complex plane, $\lambda (K)$ is the *K*-uniform distance from the complex conjugation to the algebra of all rational functions with poles outside *K*. Thus, given any integer $n\ge 1$, it is proven that each compactum *K* can be decomposed as the union of two new compact sets $E_1$ and $E_2$ with $\lambda (E_j)\le 1/n$ for $j=1,2$. Moreover, we also show that no compactum *K* with positive analytic content can be decomposed as the countable union of compact sets of zero analytic content.

## Introduction

Let $\mathcal {C}(K)$ be the algebra of all complex continuous functions *g* defined on a compact set $K\subset \mathbb {C}$ and endowed with the uniform norm $\displaystyle \Vert g\Vert _K=\max _K|g|$. Given the sub-algebra *R*(*K*) of all $g\in \mathcal {C}(K)$ which can be uniformly approximated on *K* by rational (meromorphic) functions with poles outside *K*, the analytic content $\lambda (K)$ is defined as the uniform distance from the complex conjugate function $z\mapsto \overline{z}$ to the uniform algebra *R*(*K*); i.e.,

1 $$\begin{aligned} \lambda (K)=\inf _{h\in {R}(K)} \big \Vert h(z)-\overline{z}\big \Vert _K. \end{aligned}$$

We simplify the notation by setting $\lambda (\emptyset ):=0$ and accepting that all compact sets lie in $\mathbb {C}$. Khavinson introduced the concept of analytic content on page 51 of his 1983 thesis [1], but he called it *rational capacity*; see also the articles [2] and [3]. The much better term, *analytic content*, appeared in his paper with Gamelin [4], and it stuck. Since Stone-Weierstraß’ theorem states that every function $g\in \mathcal {C}(K)$ can be uniformly approximated on the compact set *K* by polynomials of the form $p(z,\overline{z})$, the analytic content actually *measures* how different *R*(*K*) is from $\mathcal {C}(K)$. Thus, $\lambda (K)=0$ if and only if *R*(*K*) is identically equal to $\mathcal {C}(K)$. Several properties are shown in [1–5]:

- $\lambda (\xi +bK)=|b|\lambda (K)$ for all compact sets *K* and points $\xi ,b$ in $\mathbb {C}$.
- $\lambda (K_1)\le \lambda (K_2)$ for all compact sets $K_1\subset {K_2}.$
- Given any (non-trivial) compact set *E*, whose boundary $\partial {E}$ has finite one-dimensional length, one has that
  2 $$\begin{aligned} \frac{2~\hbox {area}(E)}{\hbox {length}(\partial {E})} \le \lambda (E)\le \sqrt{\hbox {area}(E)/\pi }. \end{aligned}$$
- Given any decreasing sequence $K_1\supseteq {K_2}\supseteq {K_3}\supseteq \cdots$ of compact sets,
  3 $$\begin{aligned} {\lambda \Big ({\textstyle \bigcap _{j\ge 1}}K_j \Big )=\lim _{j\rightarrow \infty }\lambda (K_j).} \end{aligned}$$

The last property was originally proved under stronger hypotheses, but the ideas presented in [1, 3] can be easily generalized to obtain a general case. For the sake of completeness, we include a proof of (3) in Lemma 2 below. On the other hand, one can calculate the analytic content of a compact disk $\mathbb {D}(\xi ,R)$ with centre at $\xi \in \mathbb {C}$ and radius $R>0$, as well as that of a compact annulus $\mathbb {A}(\xi ,R,r)$ with centre at $\xi \in \mathbb {C}$ and radii $R>r>0$. Indeed,

4 $$\begin{aligned} \lambda \big (\mathbb {D}(\xi ,R)\big )=R \quad \hbox {and}\quad \lambda \big ( \mathbb {A}(\xi ,R,r)\big )=R{-}r. \end{aligned}$$

These identities are easily obtained from (2) and the following ones,

$$\begin{aligned} \big \Vert \overline{\xi }{-}\overline{z} \big \Vert _{\mathbb {D}(\xi ,R)}\!=R\quad \hbox {and}\quad \Big \Vert \frac{rR}{z{-}\xi } +\overline{\xi }-\overline{z} \Big \Vert _{\mathbb {A}(\xi ,R,r)}\!=R{-}r. \end{aligned}$$

Thus, in the particular cases when *K* is the compact disk or annulus, one indeed knows which function $h\in {R}(K)$ minimizes the infimum in (1), and so such *h*(*z*) yields the uniform distance from *R*(*K*) to the complex conjugate function.

Considering the above-mentioned properties, one may wonder whether the analytic content $\lambda$ is also countably subadditive, so that $\lambda$ induces an outer measure on $\mathbb {C}$. This question is inspired by Tolsa’s recent results on the countable semiadditivity of the *analytic capacity*

5 $$\begin{aligned} \gamma (K)=\sup \Big \{|f'(\infty )|:f\in \mathcal {O}(\mathcal {S}^2{\setminus }K), \,\Vert f\Vert _{\mathcal {S}^2\setminus {K}} \le 1,\,f(\infty )=0\Big \}, \end{aligned}$$

where $K\subset \mathbb {C}$ is any given compact set. The functions *f* are holomorphic and bounded by one on the Riemann sphere $\mathcal {S}^2$ minus *K*. Tolsa has proved the existence of a positive constant $c_1>0$ such that

$$\begin{aligned} \textstyle \gamma \Big (\bigcup _{j\ge 1}K_j \Big )\,\le \,c_1\sum _{j\ge 1}\gamma (K_j) \end{aligned}$$

for every collection of compact sets $\{K_j\}_{j\ge 1}$ in $\mathbb {C}$; see for example [6–8]. Hence, the main objective of this work is to prove that the analytic content $\lambda (\cdot )$ is neither subadditive nor semiadditive. We indeed show that:

### Theorem 1

Given any compact set $K\subset \mathbb {C}$ and integer $n\ge 1$, there exists a pair of new compact sets $E_0$ and $E_1$ in $\mathbb {C}$ such that

6 $$\begin{aligned} K=E_0\cup {E_1}\quad \hbox {and}\quad \lambda (E_j)\le 1/n\quad \hbox {for}\quad {j}=0,1. \end{aligned}$$

This result is proved in the third section of this paper; after we show some basic necessary results in the second section. Finally, we close this work by presenting related results in section four. In particular, we prove that no compact set *K*, with positive analytic content $\lambda (K)>0$, can be decomposed as the countable union of compact sets of zero analytic content.

## Basic results

We begin presenting some known results. We include their proofs for the sake of completeness and because these results are the main reason behind the construction in Theorem 1. Thus, it may sound counterintuitive, but Theorem 1 is a direct consequence of the strong subadditivity of $\lambda (\cdot )$, for the particular case when the compact sets are disjoint.

### Lemma 1

Given two disjoint compact sets $K_1$ and $K_2$ in $\mathbb {C}$, the analytic content satisfies

7 $$\begin{aligned} {\lambda (K_1\cup {K_2})=\max \big \{ \lambda (K_1),\lambda (K_2)\big \}.} \end{aligned}$$

### Proof

Obviously, $\lambda (K_1\cup {K_2})\ge \max \big \{\lambda (K_1),\lambda (K_2)\big \}$. Hence, we only need to show the opposite inequality. Given any $\varepsilon >0$, there exists a pair of rational (meromorphic) functions $h_1$ and $h_2$, such that $h_j$ has no poles on $K_j$ and

8 $$\begin{aligned} \big \Vert h_j(z){-}\overline{z}\big \Vert _{K_j} \!<\lambda (K_j)+\varepsilon \quad \hbox {for}\quad {j}=1,2. \end{aligned}$$

Now then, since the compact sets $K_1$ and $K_2$ are disjoint, there are two disjoint open sets $U_1$ and $U_2$, such that $K_j\subset {U_j}$ and $h_j$ has no poles on $U_j$ for $j=1,2$, and so the function

$$\begin{aligned} G(z)=\left\{ \begin{array}{cl} h_1(z)& \hbox {if}~z\in {U_1},\\ h_2(z)& \hbox {if}~z\in {U_2}, \end{array}\right. \end{aligned}$$

is holomorphic on an open neighbourhood $U_1\cup {U_2}$ of the union $K_1\cup {K_2}$. The inequalities (8) can then be rewritten as follows

9 $$\begin{aligned} \big \Vert G(z){-}\overline{z}\big \Vert _{K_1\cup {K_2}} \!=\max _{j=1,2}\big \Vert h_j(z){-}\overline{z} \big \Vert _{K_j}\!<\max _{j=1,2}\lambda (K_j) +\varepsilon . \end{aligned}$$

On the other hand, Runge’s Theorem then yields a third rational function *H* with poles outside $K_1\cup {K_2}$ and such that

10 $$\begin{aligned} \big \Vert H-G\big \Vert _{K_1\cup {K_2}}<\varepsilon . \end{aligned}$$

The previous identities (9)-(10) automatically yield

$$\begin{aligned} \big \Vert H(z){-}\overline{z}\big \Vert _{K_1\cup {K_2}} \!<\max \big \{\lambda (K_1),\lambda (K_2)\big \} +2\varepsilon . \end{aligned}$$

The desired result (7) follows from (1) and the fact that $\varepsilon >0$ was taken to be arbitrary. $\square$

It is interesting to compare this result against Lemma 4 of [9, 10] or the corollary on page 583 of [11]; where it is shown that identity (7) holds, when $\lambda (K_j)$ vanishes for $j=1,2$, but $K_1\cap {K_2}$ need not be empty. Moreover, as announced in the introduction, we also prove the identity (3).

### Lemma 2

Given any decreasing sequence $K_1\supseteq {K_2}\supseteq {K_3}\supseteq \cdots$ of compact sets in $\mathbb {C}$, one has that

11 $$\begin{aligned} \lambda (\mathbb {K})=\lim _{j\rightarrow \infty } \lambda (K_j)\quad \hbox {for}\quad \mathbb {K} ={\textstyle \bigcap _{j\ge 1}}K_j. \end{aligned}$$

### Proof

Without loss of generality, one can assume that no $K_j$ is empty. Hence, $\mathbb {K}$ is also non-empty because of Cantor’s intersection theorem. Now then, given any $\varepsilon >0$, let *h* be a rational function with poles outside $\mathbb {K}$ and such that

12 $$\begin{aligned} \lambda (\mathbb {K})\le \Vert h(z){-}\overline{z} \Vert _{\mathbb {K}}<\lambda (\mathbb {K})+\varepsilon . \end{aligned}$$

Now consider the following open neighbourhood of $\mathbb {K}$,

13 $$\begin{aligned} U_\varepsilon =\big \{z\in \mathbb {C} :|h(z){-}\overline{z}|<\lambda (\mathbb {K})+\varepsilon \big \}. \end{aligned}$$

We assert that $K_N\subset {U_\varepsilon }$ for some integer $N\ge 1$. Otherwise, if all compacta $K_j$ meet the complement $\complement {U}_\varepsilon =\mathbb {C}{\setminus }U_\varepsilon$, we have a decreasing sequence of non-empty compacta:

$$\begin{aligned} (K_1\cap \complement {U}_\varepsilon )\supseteq (K_2\cap \complement {U}_\varepsilon )\supseteq (K_3 \cap \complement {U}_\varepsilon )\supseteq \cdots \end{aligned}$$

Therefore, $\mathbb {K}\cap \complement {U_\varepsilon } =\bigcap _{j\ge 1}K_j\cap \complement {U_\varepsilon }$ is not empty according to Cantor’s intersection theorem, which is a contradiction to equations (12)-(13). Thus, given $\varepsilon >0$, there is an index $N\ge 1$ such that $K_N\subset {U}_\varepsilon$; consequently

$$\begin{aligned} \mathbb {K}\subset {K_j}\subset {U}_\varepsilon \quad \hbox {and}\quad \lambda (\mathbb {K})\le \lambda (K_j)<\lambda (\mathbb {K})+\varepsilon \end{aligned}$$

for every integer $j\ge {N}$. Recall the hypotheses and construction (13). The last inequalities above yield the desired result (11). $\square$

We also need the following simple result.

### Lemma 3

Let *K* be any compact set contained in some closed rectangle $\Pi \subset \mathbb {C}$ of sides $u\ge 0$ and $w\ge 0$, then the analytic content $\lambda (K)\le \min \{u,w\}$.

### Proof

We can assume without loss of generality that $u\ge {w}$ and the rectangle $\Pi$ is centred at the origin and its sides are parallel to the main coordinated axes, so that its corners are located at the four points $\pm \frac{u}{2}\pm {i}\frac{w}{2}$. The result easily follows from the definition (1) after taking $h(z)=z$,

$$\begin{aligned} \lambda (K)\le \lambda (\Pi )\le \Vert z-\overline{z}\Vert _\Pi =w. \end{aligned}$$

$$\square $$

## Proof of Theorem 1

Recall the given hypotheses: $K\subset \mathbb {C}$ is any compact set and $n\ge 1$ is a fixed integer. Assume that *K* is contained inside a disk $\mathbb {D}(0,m)$ with centre at the origin and integer radius $m\ge 2$ large enough. We construct a pair of compact sets $E_0$ and $E_1$ such that

14 $$\begin{aligned} K=E_0\cup {E_1}\quad \hbox {and}\quad \lambda (E_j)\le 1/n\quad \hbox {for}\quad {j}=0,1, \end{aligned}$$

by considering the following closed rectangles of sides 2*m* and 1/*n*

15 $$\begin{aligned} \textstyle \Pi (k,n)=\Big \{z\in \mathbb {C} :|\Re (z)|\le {m},\,\Im (z)\in \Big [ \frac{k}{n},\frac{k+1}{n}\Big ]\Big \}. \end{aligned}$$

We take $k\in \mathbb {Z}$ to be an arbitrary integer. Lemma 3 easily implies that the analytic content $\lambda (\Pi (k,n))\le {1/n}$, because $m\ge 2$ and $n\ge 1$. The main idea here is to use the rectangles (15) to cut *K* into pieces, and then reassemble the pieces into two compact sets, on which we can apply Lemma 1. I.e., define

$$\begin{aligned} E_j=\bigcup _{k=-mn}^{mn}\!\Big (K\cap \Pi (j{+}2k,n)\Big )\quad \hbox {for}\quad {j}=0,1, \end{aligned}$$

so that *K* is equal to $E_0\cup {E_1}$. Notice that $E_0$ is the union of a finite number of disjoint compact sets, each contained inside some rectangle given in (15). Lemmas 1 and 3 automatically imply that $\lambda (E_0)\le 1/n$. The same can be said about the analytic content of $E_1$, and so the main identities (14) and (6) in Theorem 1 hold, as we wanted to show.

## Final results

Since the construction in Theorem 1 is strongly based on Lemmas 1 and 3, rather than on other specific properties of the analytic content, we can generalise this result to consider other set functions. Furthermore, we can relax the hypotheses to avoid using Lemma 3.

### Theorem 2

Let $\varphi :\mathcal {B}\rightarrow [0,\infty )$ be any set function defined on the Borel $\sigma$-algebra $\mathcal {B}$ of $\mathbb {C}$, which satisfies the properties:

1. $\varphi (\xi +bK)=|b|\varphi (K)$ for all points $\xi ,b\in \mathbb {C}$ and compact sets $K\subset \mathbb {C}$.
2. $\varphi (X_1)\le \varphi (X_2)$ for every pair of compact sets $X_1\subset {X_2}$ in $\mathbb {C}$.
3. $\varphi (K_1\cup {K_2})\le \max \{\varphi (K_1),\varphi (K_2)\}$ for all compact sets $K_1\cap {K_2}=\emptyset$.

Then, given any compact set $K\subset \mathbb {C}$ and integer $n\ge 1$, there are four new compact sets $\{E_j\}_{j=1}^4$, such that

$$\begin{aligned} \textstyle {K}=\bigcup _{j=1}^4E_j \quad \hbox {and}\quad \varphi (E_j)\le \varphi (\boxtimes )/n, \end{aligned}$$

for all $j=1,2,3,4$ and where $\boxtimes \subset \mathbb {C}$ is a square with sides of length 1.

As a direct application, one deduces that the *analytic capacity* $\gamma (\cdot )$ in (5) cannot satisfy condition 3 above. Otherwise, Theorem 2 would contradict Tolsa’s results on the semiadditivity of the analytic capacity.

### Proof of Theorem 2

We proceed as in the proof of Theorem 1 (section 3). Assume that *K* is contained inside a disk $\mathbb {D}(0,m)$ for some integer radius $m\ge 1$ large enough and construct the compact sets:

$$\begin{aligned} \Xi (j,k,n)= &   \textstyle \Big \{z\in \mathbb {C}: \Re (z)\in \Big [\frac{j}{n},\frac{j+1}{n}\Big ],\, \Im (z)\in \Big [\frac{k}{n},\frac{k+1}{n}\Big ]\Big \},\\ E_1= &   \bigcup _{j,k=-mn}^{mn}\! \Big (K\cap \Xi (2j,2k,n)\Big ),\\ E_2= &   \bigcup _{j,k=-mn}^{mn}\! \Big (K\cap \Xi (2j{+}1,2k,n)\Big ),\\ E_3= &   \bigcup _{j,k=-mn}^{mn}\! \Big (K\cap \Xi (2j,2k{+}1,n)\Big ),\\ E_4= &   \bigcup _{j,k=-mn}^{mn}\! \Big (K\cap \Xi (2j{+}1,2k{+}1,n)\Big ). \end{aligned}$$

$$\square $$

We must also point out that Theorem 1 cannot be improved by replacing the inequalities $\lambda (E_j)\le \frac{1}{n}$ in (6) with the condition that $\lambda (E_j)=0$ for $j=0,1$, because no compact set $K\subset \mathbb {C}$, with positive analytic content $\lambda (K)>0$, can be decomposed as the countable union of compact sets of zero analytic content.

### Theorem 3

Let $\{K_j\}_{j\ge 1}$ be a countable collection of compact sets in $\mathbb {C}$, whose analytic content $\lambda (K_j)=0$ for every $j\ge 1$.

16 $$\begin{aligned} \hbox {If}\quad \mathbb {K}={\textstyle \bigcup _{j\ge 1}K_j} \quad \hbox {is compact, then}\quad \lambda (\mathbb {K})=0. \end{aligned}$$

Recall that $\lambda (\mathbb {K})=0$ if and only if the algebras $R(\mathbb {K})$ and $\mathcal {C}(\mathbb {K})$ coincide. The proof follows the ideas presented in Lemma 4 of [9, 10].

### Proof

Given any $\varepsilon >0$, let $h_1$ be a rational function with poles outside $K_1$ and which satisfies

17 $$\begin{aligned} \Vert h_1(z)-\overline{z}\Vert _{K_1}<\varepsilon /2. \end{aligned}$$

Take an open bounded neighbourhood $U_2$ of $K_1$, with piecewise smooth boundary and such that: $h_1$ has no poles on the compactum $\overline{U_2}$ and

18 $$\begin{aligned} \Vert h_1(z)-\overline{z}\Vert _{\overline{U_2}} <\varepsilon /2. \end{aligned}$$

The Tietze-Urysohn-Brouwer extension theorem implies the existence of a continuous function $g_2$ defined on $\overline{U_2}\cup {K_2}$ so that

19 $$\begin{aligned} g_2|_{\overline{U_2}}=h_1|_{\overline{U_2}} \quad \hbox {and}\quad \Vert g_2(z){-}\overline{z} \Vert _{\overline{U_2}\cup {K_2}}\!<\varepsilon /2. \end{aligned}$$

Recall that $h_1|_{\overline{U_2}}\in {R}(\overline{U_2})$ and $R(K_2)=\mathcal {C}(K_2)$, because $h_1$ is rational with poles outside $\overline{U_2}$ and the analytic content $\lambda (K_2)=0$. Lemma 4 of [9, 10] yields the existence of a rational function $h_2$ with no poles on $\overline{U_2}\cup {K_2}$, which satisfies

20 $$\begin{aligned} \textstyle \Vert h_2-g_2\Vert _{\overline{U_2}\cup {K_2}} \!<\frac{\varepsilon }{4}\quad \hbox {and}\quad \Vert h_2 (z){-}\overline{z}\Vert _{\overline{U_2}\cup {K_2}} \!<{\frac{3}{4}}\varepsilon . \end{aligned}$$

If we repeat the analysis done in the paragraphs above, using the last identity in (20) instead of (17), we can construct both an open bounded neighbourhood $U_3$ of the union $\overline{U_2}\cup {K_2}$ and a rational function $h_3$ with poles outside the compactum $\overline{U_3}\cup {K_3}$, in such a way that

$$\begin{aligned} \Vert h_3(z)-\overline{z}\Vert _{\overline{U_3}\cup {K_3}} <7\varepsilon /8. \end{aligned}$$

Furthermore, we can inductively build an increasing sequence of open sets

21 $$\begin{aligned} \emptyset =U_1\subset {U_2}\subset {U_3}\subset \cdots \end{aligned}$$

and a sequence of rational functions $\{h_n\}_{n\ge 1}$, which satisfy

22 $$\begin{aligned} \textstyle \overline{U_n}\cup {K_n}\subset U_{n+1}\quad \hbox {and}\quad \Vert h_n(z){-} \overline{z}\Vert _{\overline{U_n}\cup {K_n}} \!<\frac{2^n-1}{2^n}\varepsilon , \end{aligned}$$

for all indices $n\ge 1$. Obviously, every $h_n$ has its poles outside $\overline{U_n}\cup {K_n}$. Finally, the increasing sequence in (21) is an open cover of the compactum $\mathbb {K}$ in (16), because of the first part of (22). Therefore, there exists an integer $N\ge 1$ such that $\mathbb {K}\subset {U_N}$, and so the second part of (22) also yields

$$\begin{aligned} \Vert h_N(z)-\overline{z}\Vert _{\mathbb {K}}<\varepsilon . \end{aligned}$$

One can conclude that $\lambda (\mathbb {K})=0$ because $\varepsilon >0$ is arbitrary. $\square$

We must point out that the existence of the rational function $h_2$ in (20) is also a consequence of Bishop’s results, as cited in Lemma 11.7 and Theorem 11.8 of [12]. We only need to request that the function $g_2$ in (19) be continuous on the whole Riemann sphere and holomorphic (equal to $h_1$) on a small neighbourhood of the compactum $\overline{U_2}$. Otherwise, we can also build $h_2$ in (20) via the corollary on page 583 of [11]. We only need to request that the open bounded neighbourhood $U_2$ of $K_1$ in (18) have instead piecewise linear boundary; i.e., $\partial {U}_2$ is composed by a finite number of line intervals. Thus, $\partial {U}_2$ and the set $\partial {U}_2{\cap }\partial {K_2}$ are both analytically negligible; see page 583 of [11] or section VIII-13 of [13].

The proof of Theorem 3 can be adapted to show the partial subaditivity of the analytic content.

### Lemma 4

Let $E_1$ and $E_2$ be a pair of compact sets in $\mathbb {C}$.

23 $$\begin{aligned} \hbox {If}\quad \lambda (E_2)=0,\quad \hbox {then} \quad \lambda (E_1\cup {E_2})=\lambda (E_1). \end{aligned}$$

### Proof

We only need to prove that $\lambda (E_1\cup {E_2})\le \lambda (E_1)$. Given any $\varepsilon >0$, let $h_1$ be a rational function with poles outside $E_1$ and which satisfies

$$\begin{aligned} \lambda (E_1)\le \Vert h_1(z)-\overline{z} \Vert _{E_1}<\lambda (E_1)+\varepsilon . \end{aligned}$$

Working as in (18) and (19), we deduce the existence of an open neighbourhood $U_2$ of $E_1$ and of a continuous function $g_2$ defined on $\overline{U_2}\cup {E_2}$ so that:

- $g_2|_{\overline{U_2}}=h_1|_{\overline{U_2}}$ lies in $R(\overline{U_2})$, since it is rational with poles outside $\overline{U_2}$.
- The inequality $\Vert g_2(z)-\overline{z}\Vert _{\overline{U_2}\cup {E_2}} \!<\lambda (E_1)+\varepsilon$ holds.

Recall that $R(K_2)=\mathcal {C}(K_2)$, because the analytic content $\lambda (K_2)=0$. Lemma 4 of [9, 10] implies that $g_2$ lies in $R(\overline{U_2}\cup {E_2})$. Hence, there exists a rational function $h_2$ with no poles on $\overline{U_2}\cup {E_2}$, which satisfies

$$\begin{aligned} \textstyle \Vert h_2-g_2\Vert _{\overline{U_2}\cup {E_2}}\!< \varepsilon \quad \hbox {and}\quad \Vert h_2(z){-}\overline{z} \Vert _{E_1\cup {E_2}}\!<\lambda (E_1){+}2\varepsilon . \end{aligned}$$

The desired result (23) follows from (1) and the fact that $\varepsilon >0$ was taken to be arbitrary. $\square$

We close this work by presenting a counterexample to show that the compactness of $\mathbb {K}$ cannot be removed from the hypotheses in Theorem 3.

### Example 1

Take the following subsets of the real axis in $\mathbb {C}$:

$$\begin{aligned} K_j=[-1,-1/j]\cup [1/j,1]\quad \hbox {for any integer}\quad {j}\ge 1. \end{aligned}$$

One has that all $\lambda (K_j)=0$ and $R(K_j)=\mathcal {C}(K_j)$. However, the set

$$\begin{aligned} \textstyle {B}=\bigcup _{j\ge 1} K_j=[-1,0)\cup (0,1] \end{aligned}$$

is non-compact and the algebras $R(B)\ne \mathcal {C}(B)$, because the function $\sin (1/z)$ is indeed continuous on $B\subset \mathbb {R}$, but it cannot be uniformly approximated on *B* by rational functions with poles outside *B*.

## Data Availability

No datasets were generated or analysed during the current study.
